# Is It Time to Abandon the Kidney-Centered View on the Origin of Primary Hypertension?

**DOI:** 10.1161/HYPERTENSIONAHA.125.24002

**Published:** 2025-08-05

**Authors:** Liffert Vogt, Vitória Cunha, Anna F. Dominiczak, Guido Grassi, Marek Rajzer, Agostino Virdis, Thomas Weber

**Affiliations:** Department of Internal Medicine, Section Nephrology, Amsterdam UMC, Location AMC, the Netherlands (L.V.).; Amsterdam Cardiovascular Sciences, University of Amsterdam, the Netherlands (L.V.).; Internal Medicine, MD, Unidade Local de Saúde Almada Seixal/Hospital Garcia de Orta, Portugal (V.C.).; School of Cardiovascular and Metabolic Health, University of Glasgow, Scotland, United Kingdom (A.F.D.).; Department of Medicine and Surgery, University Milano Bicocca, Italy (G.G.).; First Department of Cardiology, Interventional Electrocardiology, and Arterial Hypertension, Jagiellonian University Medical College, Krakow, Poland (M.R.).; Department of Clinical and Experimental Medicine, University of Pisa, Italy (A.V.).; Cardiology Department, Klinikum Wels-Grieskirchen, Austria (T.W.).

**Keywords:** blood vessels, exposome, hypertension, immune system, kidney

## Abstract

Traditionally, the kidney has been thought to play a key role in the development of hypertension. Disturbed sodium regulatory pathways can lead to primary hypertension, with abnormalities in the pressure-natriuresis mechanism contributing to its onset. An adverse intrauterine environment and postnatal stressors can affect nephron number, further linking renal development to hypertension risk. The development of hypertension may, however, also be influenced by alternative factors beyond the kidney. Monogenic diseases and polygenic risk scores are associated with hypertension development. Epigenetic mechanisms can influence the phenotype of the vascular endothelium in response to environmental stimuli, potentially leading to changes in blood pressure. Regulation of vascular tone, microvascular rarefaction, and interactions with the immune system are other nonrenal factors contributing to hypertension. The exposome, including air pollution and noise, has its impact already before conception via maternal and paternal influences, as well as later in life. In addition, lifestyle factors such as sodium and alcohol intake and tobacco use have been linked to hypertension through mechanisms such as increased sympathetic activity and vasoconstriction, highlighting the importance of behavioral factors in hypertension development. Age-related stiffening becomes important in later life, influences blood pressure phenotype, and may even precede hypertension development. Considering these multiple contributors, relevant for pathophysiology, prevention, and management of hypertension, the question arises whether the kidney-centered view on hypertension is sufficient or whether a more integrative, multifactorial perspective is needed. Full understanding of renal and nonrenal factors and their interactions driving hypertension is crucial to curb the global burden of this disease.

In 1898, Bergman and Tigerstedt^1^ demonstrated that infusions of renal homogenates, attributed to the presence of the renal hormone renin, elevated blood pressure (BP) in rabbits, there by establishing the foundational role of renal mechanisms in hypertension. In addition, low nephron endowment—arising from impaired intrauterine renal development, low birth weight, or postnatal renal tissue loss—has long been identified as a hypertension risk factor.^2^ Autopsy studies reveal fewer glomeruli in hypertensive individuals compared with normotensive controls.^2^ Other landmark experimental data by Goldblatt^3^, and confirmed in humans, showed that when a hypertensive kidney was transplanted into a normotensive recipient, the recipient developed hypertension. Conversely, after removal of a hypertensive kidney or transplantation of a normotensive kidney into a hypertensive animal, BP often returned to normal. These data led to the recognition of 2 key pathways in hypertension pathogenesis: the renin-angiotensin-aldosterone system (RAAS) and nephron endowment. The RAAS, triggered by renal renin release, drives a cascade leading to angiotensin II production—a potent vasoconstrictor that promotes vasoconstriction, sodium retention, and aldosterone secretion, all contributing to elevated BP. Intrarenal renin-angiotensin system activity amplifies these effects, reinforcing the kidney’s role in hypertension progression. Simultaneously, reduced nephron endowment prompts compensatory mechanisms like glomerular hyperfiltration and enlargement, which can cause long-term hypertensive damage and progressive renal decline, perpetuating BP elevation. These mechanisms are exacerbated by high dietary salt intake, providing evidence for the concept that high BP elevations are needed to elicit a pressure-natriuresis response for restoration of the sodium balance.^4^

The kidney-centered understanding of primary hypertension has been particularly prevalent among nephrologists, and indeed strategies such as reducing salt intake, thiazide diuretics, and RAAS blockade have proven highly effective in lowering BP.^5^ However, old—as proposed almost 60 years ago by Page^6^—and newly uncovered nonrenal factors (summarized in Figure 1 and reference^7^) challenge the sufficiency of this view, confirming a need for a more integrative, multifactorial perspective on hypertension. This review systematically explores emerging data that may redefine our understanding of primary hypertension pathophysiology.

**Figure 1.**
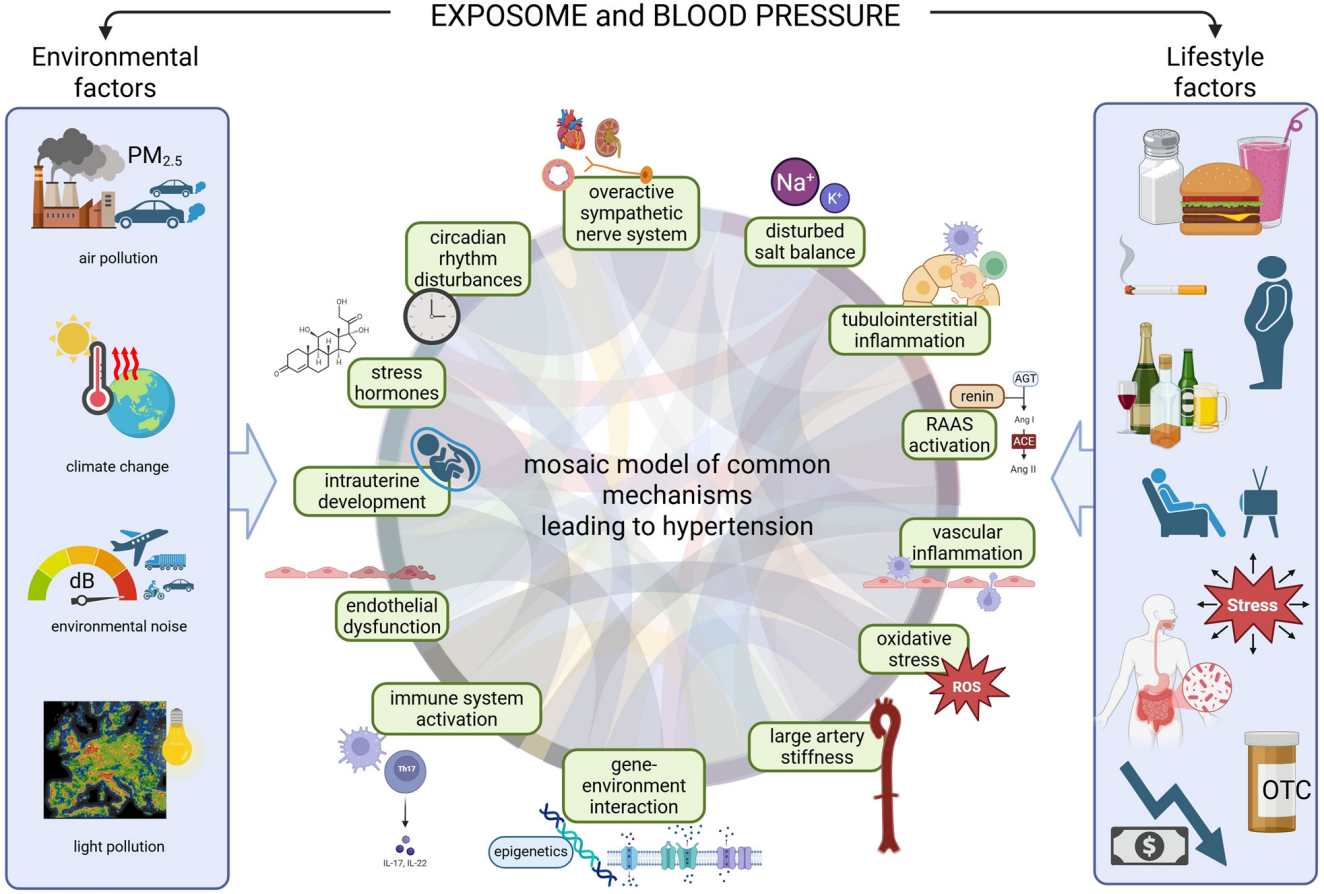
**Overview of how the exposome, encompassing both external environmental and lifestyle factors, interacts with well-established and novel pathomechanisms in the development of hypertension.** While many of these factors cannot be seen fully independent from each other and their action to the kidney, obscuring clear cut cause-effect relationships, they generally also influence the heart and vasculature, including vascular tone, capillary densities, and endothelial integrity. dB indicates decibel; OTC, over-the-counter; PM_2.5_, particulate matter ≤2.5 µm in diameter; RAAS, renin-angiotensin-aldosterone system; and ROS, reactive oxygen species.

## Genetic Factors

**Figure 2.**
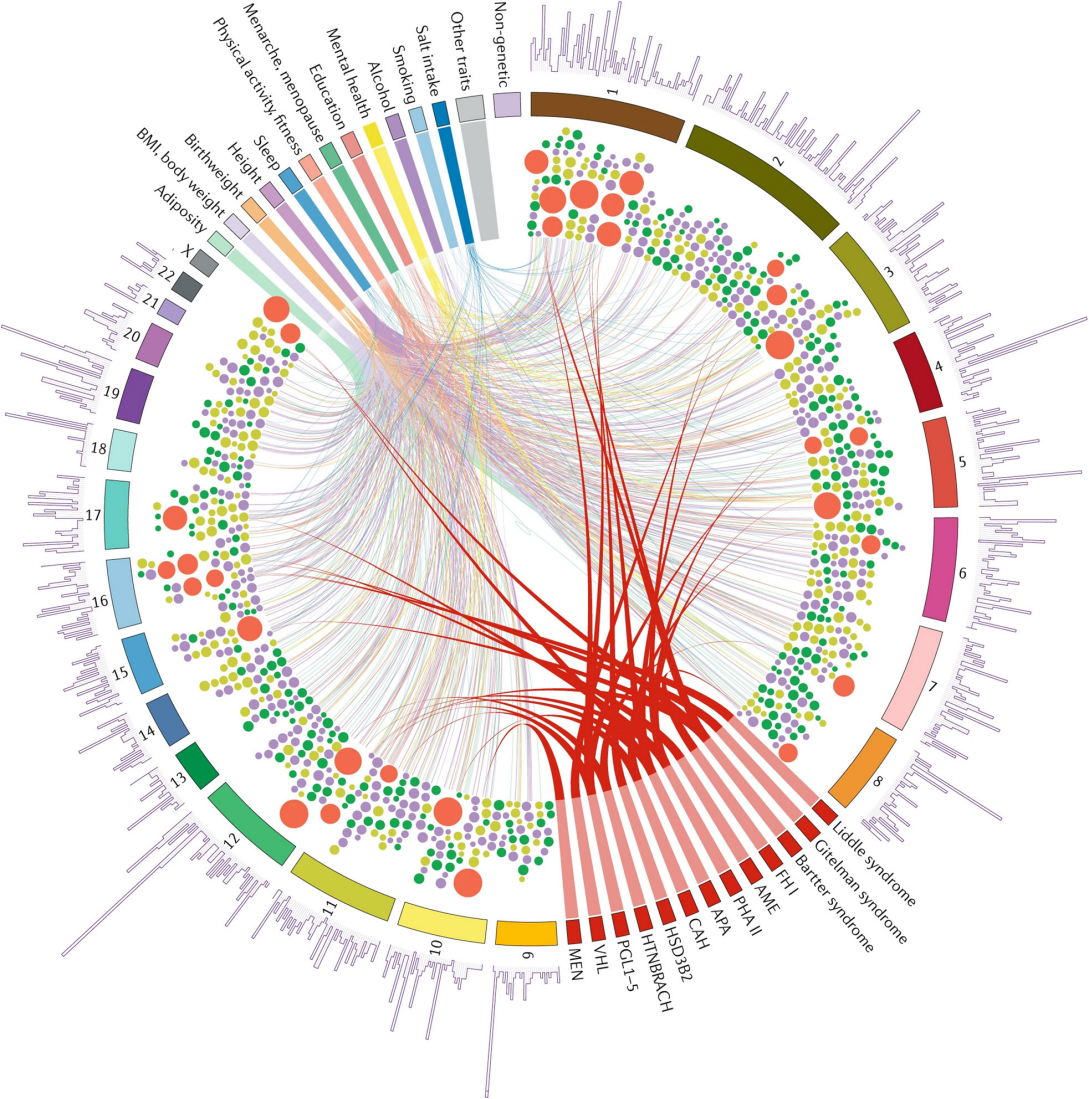
**The Circos plot, adapted and printed with permission from reference,^8^ illustrates genetic variants associated with both monogenic and polygenic disorders, as identified through linkage studies, sequencing, and genome-wide association studies (GWAS).** Genetic variants associated with monogenic disorders are represented by large filled red circles and are linked to their corresponding clinical syndromes, shown in the lower part of the circle. Polygenic variants, identified through GWAS, are shown as smaller circles in purple, dark green, and light green, representing associations with systolic blood pressure, diastolic blood pressure, and pulse pressure, respectively. Chromosomes are displayed as numbered segments. The top of the circle highlights pleiotropic signals from phenome-wide association studies, which reflect lifestyle, environmental, and early life influences on blood pressure, mapped to the locations of pleiotropic SNPs. The outer-ring histogram represents the number of pleiotropic associations for SNPs at each locus; taller bars indicate greater pleiotropy. APA indicates aldosterone-producing adenoma; AME, apparent mineralocorticoid excess; CAH, congenital adrenal hyperplasia; FH, familial hyperaldosteronism; GWAS, genome-wide association studies; HSD3B2, 3β-hydroxysteroid dehydrogenase deficiency; HTNBRACH, hypertension with brachydactyly; MEN, multiple endocrine neoplasia type II. PGL, paraganglioma; PHA, pseudohypoaldosteronism type II; VHL, Von Hippel-Lindau syndrome.^8^

The genetic architecture of BP includes 31 genes with rare variants causing monogenic syndromes of high and low BP and more than 2100 common single nucleotide polymorphisms (SNPs) associated with BP traits in genome-wide association studies (GWAS; Figure 2).^8^ It is of interest that all but one of the causal genetic variants in monogenic hypertension and hypotension work via pathophysiological pathways located in the kidneys and adrenal glands and relate to disturbed renal sodium handling and catecholamine excess causing high BP.^8^ Monogenic BP disorders involve not only germline but also somatic mutations, as can been seen in patients with neoplasms causing hypertension, such as phaeochromocytomas.^9^

The GWAS have contributed to the understanding of the common polygenic hypertension, but the effect size of each SNP (of the total of >2100 identified) on BP phenotype is relatively small—that is, ≈1 mm Hg for systolic BP and 0.5 mm Hg for diastolic BP. In combination, these effects are much greater. It should be noted that few SNPs identified in GWAS have been fully proven to be causal for BP and hypertension, since the majority reside in the noncoding regions of the genome. One of the exceptions with proven causality is the minor allele of an SNP (rs13333226) in the promoter region of the gene encoding uromodulin. Strong evidence from GWAS and transgenic experiments exists that the gene encoding uromodulin, exclusively expressed in the renal tubule, is involved in BP regulation through its interaction with the sodium-potassium-chloride cotransporter.^10,11^ Mendelian randomization studies support this causal role for the gene encoding uromodulin on BP. This data led to the first clinical trial validating this GWAS-identified SNP, demonstrating the potential of genotype-guided hypertension management, in this case with loop diuretics.^12^ Alternative approaches, such as candidate genes studies benefiting from GWAS data, have also identified SNPs that are related to BP—supportive to newly uncovered mechanisms not involving the kidney. A recent gene-environment analysis in 3 large cohorts, including study of over 50 000 genomic variants in the glycogenome, demonstrated that 3 variants in heparan sulfation genes were linked to high dietary sodium-mediated BP.^13^

As the genetic makeup of an individual is stable from birth, this information could be used for risk prediction. This has led to the development of polygenic risk scores, where hundreds or thousands of SNPs are aggregated to derive a single value estimate of an individual’s genetic risk of a phenotype such as hypertension or cardiovascular disease.^14^ These polygenic risk scores are particularly useful in younger people to guide early cardiovascular prevention. It has been shown in a randomized controlled study that subjects with high polygenic risk scores respond well to personalized preventative intervention and achieve significant reduction in cardiovascular risk.^15^ As our understanding of the genomics of hypertension and other complex polygenic traits increases, new opportunities to translate and integrate genomics into our clinical practice and preventative interventions will support the paradigm of precision population health.

## Microvasculature and Resistance Vessels

Microvascular damage, generally defined as endothelial dysfunction, microvessel wall structural remodeling, and microvascular rarefaction, occurs early in patients with hypertension, even at subclinical stages before the disease onset.^16^ Besides that microvascular damage has prognostic impact in terms of an elevated cardiovascular and renal risk, it is also suggested to cause high BP development.^17^

In the classical view, the peripheral microcirculation has the dual role of master regulator of systemic hemodynamics and major metabolic player, modulating the vascular tone in response to tissue metabolic demand.^16,18^ In the contemporary view, the ubiquitous microvascular endothelium represents the interface between the milieu intérieur and external stimuli. As such, it is the first target of the exposome (vide infra) and cardiometabolic risk factors. Microvascular alterations occur very early in disease pathogenesis, leaving epigenetic signatures that persist after removal of risk factors. In an epigenetically driven landscape, such as cardiovascular-kidney-metabolic disease, the microcirculation is specifically linked to hypertension already at a young age.^19^ The compromised microcirculation further promotes local and systemic damage via proinflammatory responses, not limited to the vessel^20^; other key players are namely also involved, including perivascular adipose tissue and neural terminations.^21^ Several immune cells participate in this response, including T lymphocytes (eg, Th1, Th2, Th17, Treg), neutrophils, macrophages, and dendritic cells.^20,22^ The imbalance between the subpopulations of T lymphocytes—influenced by gut microbiota adaptations^23^—leads to increased levels of proinflammatory cytokines as interferon-γ, IL (interleukin)-17, and TNF-α (tumor necrosis factor-α). Neutrophils show furthermore an increased neutrophil extracellular trap production in response to Ang II (angiotensin II), while macrophages and dendritic cells increased expression of Ang II-sensitive toll-like receptors leading to IL-1β, IL-6, and TNF-α hypersecretion. These immune-related phenomena might prove to be relevant for hypertension development since they lead to reduced nitric oxide bioavailability and microvascular remodeling.^20,24^ In addition, randomized controlled data indicate that heparan sulfate supplementation may lower BP due to restoration of endothelial integrity and via microvascular sodium binding.^25^

Focusing on the kidney, the same bidirectional relationship exists, as microvascular damage in hypertensive patients is also present in the renal microcirculation. Renal microvascular dysfunction leads to loss of renal function, usually preceded by albuminuria.^26–28^ Subsequently, chronic kidney disease may further contribute to high BP, not only via abnormalities in the pressure-natriuresis mechanism, but also due to tubulointerstitial inflammation related to loss of peritubular capillary integrity. Both CD8+T cells and the Th17-IL-17 axis are, for instance, linked to hypertension development as a result of their interaction with tubular cells and subsequent sodium-chloride cotransporter activation.^29^

In conclusion, microvasculature is suggested to be a key player in the natural history of hypertension. However, the majority of this evidence is associative. The precise notion of the temporal onset of microvascular damage is still lacking, and the different mechanisms involved in the onset of microvascular dysfunction in essential hypertension alone, or in the presence of other cardiometabolic diseases (eg, obesity), are still elusive, calling for further dedicated studies in this area.

## Exposome

The exposome generally encompasses the totality of environmental and lifestyle factors, including diet, pollution, stress, physical activity, and socioeconomic status, that interact with an individual’s biology and have a growing impact on hypertension development (Figure 1). To start with the environment, the strongest epidemiological evidence of the effects of environmental pollution on the incidence and prevalence of hypertension relates to air and noise pollution, but negative effects have also been confirmed for light pollution and climate change.^30^ In the global PURE study (Prospective Urban and Rural Epidemiological), every 10 mg/m^3^ increase in PM_2.5_ (particulate matter ≤2.5 µm in diameter—ie, the most harmful fraction of air pollution) was associated with a 4% increased risk of hypertension.^31^ This association demonstrated nonlinearity and was strongest for the fourth (PM_2.5_ >62 μg/m^3^) compared with the first (PM_2.5_ <14 μg/m^3^) quartiles (odds ratio, 1.36), whereas the currently acceptable average limit value for the European Union has been set at 25 µg/m^3^ and policy is aiming to reduce this value to 10 µg/m³ by 2030 in line with World Health Organization recommendations.^31^ In a large Chinese study, a total population-attributable fraction of 23% for long-term exposure to PM_2.5_ for hypertension incidence was calculated and this association remained significant after multiple adjustments.^32^

In addition to air pollution, more than 20% of the European population is exposed to environmental noise levels harmful to health (>55 dB of L_DEN_), most commonly caused by road traffic noise, which affects 113 million European citizens according to European Environment Agency Reports. It is estimated that only in Europe 900 000 cases of hypertension are caused by noise each year.^33^ In a meta-analysis performed by World Health Organization experts, the relative risk of hypertension prevalence increased by 5% for each 10 dB (L_DEN_) increment of road traffic noise.^34^ In a more recent analysis of the UK Biobank, hypertension incidence increases by 13% when road traffic noise rose from 55 to 65 dB of L_DEN_.^35^

The above discussed components of the external exposome, also including light pollution and climate change, are marked by common yet complex pathomechanisms (summarized in Figure 2), leading not only to hypertension but also to increased risk of cardiovascular-kidney-metabolic diseases, such as diabetes, atherosclerosis, and microvascular disease.^30^ Typically, sympathetic overactivity relate to BP elevations. Considering that PM_2.5_ elicits tubular damage and a proinflammatory phenotype of tubular cells, leading to tubulointerstitial inflammation, also renal ischemic mechanisms contributing to both sympathetic overactivity and disturbed sodium handling are presumably involved.^36,37^ The 2 main solutions proposed in the Zero Pollution Action Plan 2030 include: (1) improving air quality to reduce the number of deaths caused by air pollution by 55%; and (2) reducing the share of people chronically disturbed by transport noise by 30%—that is, measures that are expected to significantly contribute to better BP control in the population.^38^

## Lifestyle Factors

Individual lifestyle reflects a person’s, group’s, or society’s usual way of living, encompassing factors like economic status, attitudes, behaviors, and habits. While economic level may not be an individual’s direct responsibility, it influences access to health care, medications, and BP monitoring devices.^39^ Attitudes and habits—such as adherence to consultations, medication, diet, salt and alcohol intake, exercise, smoking, and belief in science—play a critical role in health outcomes, including the risk of developing hypertension (Figure 1).^40^ Though individuals hold the power to choose their lifestyle, health professionals should therefore guide patients by leveraging their knowledge and ability to influence. The position article compiled by an international panel of experts convened by the International Society of Hypertension College of Experts from 18 countries underscores that lifestyle changes represent the cornerstone of hypertension prevention and management, crucial for improving cardiovascular and renal health, and optimizing pharmacological treatments’ effects.^40^ These changes are often difficult to implement and maintain, as environments are not always conducive to healthy habits, and clinicians may be poorly rained to communicate and influence adequately, especially in the context of different cultures, ethnicities, beliefs and literacy levels.

Sodium intake is a major dietary risk factor for hypertension. High dietary sodium levels lead to impaired renal sodium excretion, increased extracellular volume, high systemic vascular resistance, microbiomal changes, renal and systemic inflammation, and activation of the RAAS, all contributing to hypertension.^41^ Other lifestyle measures—such as weight management, physical activity, balanced nutrition, and moderation of potassium, sugar, fiber, alcohol, and energy drinks—have additive benefits. Stress management, mindfulness, sleep quality, smoking cessation, pollution reduction (vide supra), and appropriate use of nonprescription medicines, such as non-steroidal anti-inflammatory drugs and licorice, and supplements also play roles in preventing hypertension. Behavioral interventions and the increasing availability of digital health tools and wearables can further personalize and sustain lifestyle-changing efforts. Besides improvement of sodium handling, lifestyle factors are effective in control of BP by ameliorating visceral fat accumulation, insulin resistance, endothelial dysfunction, oxidative stress, inflammation, and autonomic dysregulation.^40^ Like adherence to antihypertensive medication, addressing lifestyle begins with the individual. To improve BP control globally, greater emphasis is still needed on educating patients, refining clinician communication, and addressing both renal and systemic mechanisms through sustainable lifestyle changes.

## Sympathetic Nervous System

Substantial evidence suggests that primary hypertension is accompanied by sympathetic nervous system (SNS) activation through multiple studies.^42^ Indeed, studies using drugs that block cardiac sympathetic effects have shown that a significant subset of borderline hypertensive patients exhibit increased neuroadrenergic drive, evidenced by elevated heart rates.^43^ Then, meta-analyses of over 100 studies report that, despite some negative findings, venous plasma norepinephrine—an indirect marker of sympathetic tone—is significantly elevated in hypertensive compared with normotensive individuals.^44^ Furthermore, by subtracting norepinephrine clearance from norepinephrine plasma values, Australian researchers demonstrated increased norepinephrine spillover from sympathetic neuroeffector junctions in primary hypertension, particularly in younger patients.^45^ This is true particularly at the level of the kidney, the heart, and the central nervous system—that is, in 3 cardiovascular areas of major importance in homeostasis, regulation of circulation as well as in the pathogenesis of hypertension.^42^ Finally, direct intraneural microneurographic recordings of postganglionic efferent sympathetic nerve activity to skeletal muscle confirm the crucial role of sympathetic overactivity in hypertension, which may not be limited to primary forms but also to hypertension secondary to primary aldosteronism, renal artery stenosis, and BP-amplifying conditions like obesity.^42^

Synthesizing the results obtained via the various methodological approaches mentioned above, the role of the sympathetic nervous system in hypertension development can be summarized as follows. First, the sympathetic activation occurring in primary hypertension is an early phenomenon in the clinical course of the disease detectable in different BP phenotypes, including hypertension of the elderly and pregnancy-induced hypertension.^46,47^ Second, the neurogenic alteration parallels the disease progression, becoming more evident from mild to moderate and more severe hypertensive states, including therapy-resistant hypertension and malignant hypertension.^46^ Third, while sympathetic overactivity is generally not observed in most secondary forms of hypertension, exceptions include cases of renal artery stenosis and end-stage kidney disease, where SNS hyperactivity has been shown to sustain hypertension despite intensive antihypertensive measures.^46^ Finally, SNS overactivation contributes to the development and progression of target organ damage, particularly affecting the heart and kidneys. Specifically, kidney dysfunction can amplify adrenergic activation, which, in turn, exerts deleterious effects that accelerate renal disease progression. In extreme hypertensive states, including malignant hypertension, renal ischemia caused by increased sympathetic outflow to the renal vasculature—leading to vasoconstriction, endothelial dysfunction, and tubulointerstitial injury—activates the RAAS and triggers reflex sympathetic activation.^48^ This creates a vicious cycle in which ischemia further enhances SNS activity, exacerbating renal vasoconstriction and damage.

These bidirectional potentiating relationships represent the target for the favorable BP lowering effects of the therapeutic interventions, including bilateral renal nerve ablation.^42,49^ However, considering the highly variable BP-lowering efficacy of renal nerve ablation, targeting SNS overactivation caused by mechanisms outside the kidney may prove it potency in situations of uncontrolled or therapy-resistant hypertension.

## Stiff Large Arteries

Almost by definition, large arteries are at the base of hypertension, given that BP measurement is performed at this level. From a hemodynamic point of view, BP can be considered as pressure fluctuations (pulse pressure) around a certain BP level (mean arterial pressure), with mean arterial pressure determined by cardiac output and peripheral resistance (ie, the sum of small arteries and microcirculation), and pulse pressure determined by stroke volume (cardiac function) and large artery stiffness (LAS), subsequently changing wave reflections. LAS is assessed as pulse wave velocity, typically along the carotid-femoral or brachial-ankle pathway.

While increased LAS has been viewed traditionally as a consequence of elevated BP over a longer time period, recent evidence clarified that increased LAS is also a precursor of hypertension, creating a vicious cycle. Specifically, various longitudinal studies,^50–55^ starting at ages from 17.7 to 62.4 years, consistently showed that pulse wave velocity measured at baseline is a significant predictor of BP 2.2 to 7 years later, whereas the opposite—that is, BP at baseline predicting arterial stiffness at the follow-up investigation—is not always the case.^56^ Along the same lines, isolated systolic hypertension, traditionally considered the main hypertension phenotype in elderly individuals due to increased LAS, can be present in young people, with increased LAS as a major contributor as well.^57,58^

Rather than viewing large artery function in isolation, it is increasingly evident that large and small arteries, along with the microcirculation, are intricately interconnected, forming a detrimental feedback loop in hypertension.^59^ Increased LAS increases pulse pressure, wave reflections, and the transmission of pulsatile energy into small arteries and the microcirculation. These phenomena disproportionately affect high-flow, low-impedance organs, such as the brain and kidneys, leading to subclinical damage, functional impairment, and eventually clinical complications.^60–62^ Impaired renal function and uremia drive metabolic, inflammatory, and endocrine changes, including those related to chronic kidney disease-mineral bone disease. These changes promote the progression of LAS, perpetuating the vicious cycle. Furthermore, the heart is particularly vulnerable to increased LAS due to associated changes in pulsatile afterload, the timing of wave reflections, and diastolic perfusion. These alterations can lead to clinical consequences such as heart failure and ischemia.^63,64^ This interplay highlights the systemic impact of LAS and the complex interdependence between BP-mediated vascular, cardiac, and renal health.

## Integration

**Figure 3.**
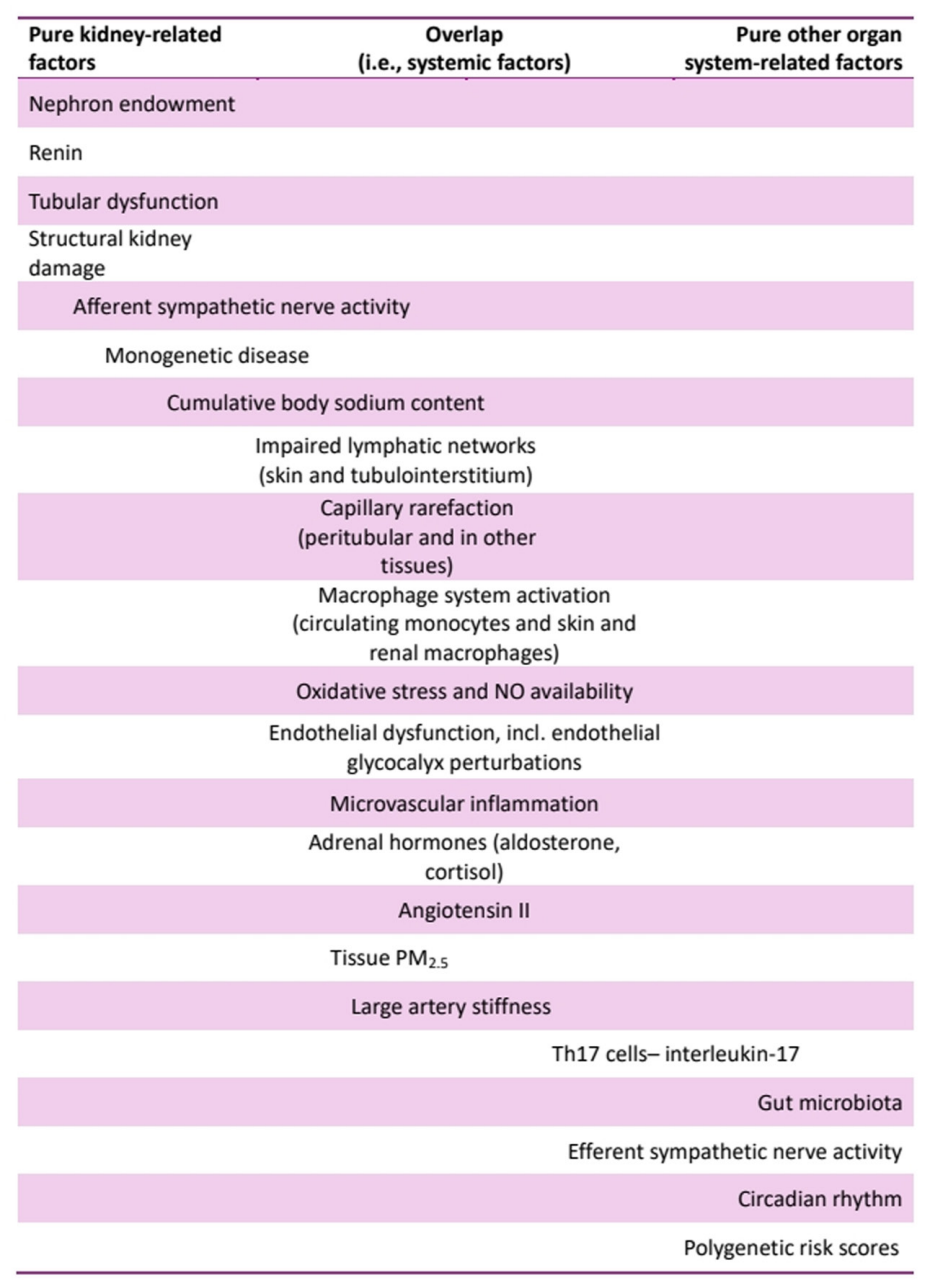
**Summary of pure renal (on the left) and (nonrenal factors) on the right contributing to hypertension development.** The placement of each factor within the table indicates potential overlap with other organ systems or a systemic action. NO indicates nitric oxide.

In this review, we examined emerging factors crucial to the development of primary hypertension (Figure 3) and explored whether they challenge the favored by nephrologists kidney-centered view of BP regulation. Across the spectrum of contributors to hypertension—genetic, epigenetic, microvascular, exposomal, SNS and large artery-related mediators—the current literature consistently demonstrates intricate, mostly reciprocal relationships with the kidney. Most associations indicate that the kidney plays an essential role in BP regulation. However, as many nonrenal factors develop in tandem with or exacerbate typical renal contributors, including nephron loss and activation of the RAAS, a solely kidney-focused view of BP regulation is incorrect. In fact, this perspective may even allow us to reframe the action of many antihypertensive measures, which are clinically viewed as kidney-targeted interventions. For instance, thiazides may lower BP over a prolonged period primarily through their vasodilatory rather than natriuretic effects—a mechanism often overlooked but critical to their efficacy.^65^ Similar observations apply to the BP-lowering effects of dietary salt restriction and RAAS blockade, which likely influence both fluid volume-mediated renal and—perhaps predominantly—vascular pathways.^41^ Nevertheless, the extent to which many of these effects occur fully independent of renal function remains to be defined.

## Article Information

### Acknowledgments

This contribution stems from the controversy debate held at the 2024 annual European Society of Hypertension meeting in Berlin, titled Why Do People Develop Primary Hypertension?, in which all contributing authors participated.

### Sources of Funding

L. Vogt reported receiving funding from Dutch Kidney Foundation (Kolff grant number: 18OKG12), CSL Vifor, Bayer, AstraZeneca, and receiving personal fees from Mineralys Therapeutics and Boehringer Ingelheim paid to his institution. A.F. Dominiczak reported receiving funding from The Living Laboratory for Precision Medicine (United Kingdom Research and Innovation Strength in Places Fund; SIPF00007/1). T. Weber reported lecture and adboard fees from Bayer, Servier, AstraZeneca, Medtronic, Merck, Gedeon Richter, Boehringer Ingelheim, ReCor, and Pfizer.

### Disclosures

None.
